# Does the relative importance of the OxCAP-MH’s capability items differ according to mental ill-health experience?

**DOI:** 10.1186/s12955-022-02009-6

**Published:** 2022-06-24

**Authors:** Timea Mariann Helter, Alexander Kaltenboeck, Josef Baumgartner, Franz Mayrhofer, Georg Heinze, Andreas Sönnichsen, Johannes Wancata, Judit Simon

**Affiliations:** 1 Department of Health Economics, Center for Public Health, Medical University of Vienna, Kinderspitalgasse 15, 1090 Vienna, Austria; 2 Department of Psychiatry and Psychotherapy, Clinical Division of Social Psychiatry, Medical University of Vienna, Währinger Gürtel 18-20, 1090 Vienna, Austria; 3 Primary Healthcare Center Medizin Mariahilf, Mariahilfer Straße 95, 1060 Vienna, Austria; 4 Department of General Practice and Family Medicine, Center for Public Health, Medical University of Vienna, Kinderspitalgasse 15, 1090 Vienna, Austria; 5 Institute of Clinical Biometrics, Center for Medical Statistics, Informatics and Intelligent Systems, Medical University of Vienna, Spitalgasse 23, 1090 Vienna, Austria; 6 Department of Psychiatry, University of Oxford, Warneford Hospital, Oxford, OX3 7JX UK; 7 Health Economics Research Centre, Nuffield Department of Population Health, University of Oxford, Old Road Campus, Oxford, OX3 7LF UK

**Keywords:** Capabilities, OxCAP-MH, Mental health, Economic evaluation, Value set, Preference, Best–worst-scaling, Hierarchical Bayes estimation

## Abstract

**Background:**

Some capability dimensions may be more important than others in determining someone’s well-being, and these preferences might be dependent on ill-health experience. This study aimed to explore the relative preference weights of the 16 items of the German language version of the OxCAP-MH (Oxford Capability questionnaire-Mental Health) capability instrument and their differences across cohorts with alternative levels of mental ill-health experience.

**Methods:**

A Best–Worst-Scaling (BWS) survey was conducted in Austria among 1) psychiatric patients (direct mental ill-health experience), 2) (mental) healthcare experts (indirect mental ill-health experience), and 3) primary care patients with no mental ill-health experience. Relative importance scores for each item of the German OxCAP-MH instrument were calculated using Hierarchical Bayes estimation. Rank analysis and multivariable linear regression analysis with robust standard errors were used to explore the relative importance of the OxCAP-MH items across the three cohorts.

**Results:**

The study included 158 participants with complete cases and acceptable fit statistic. The relative importance scores for the full cohort ranged from 0.76 to 15.72. Findings of the BWS experiment indicated that the items *Self-determination* and *Limitation in daily activities* were regarded as the most important for all three cohorts. *Freedom of expression* was rated significantly less important by psychiatric patients than by the other two cohorts, while *Having suitable accommodation* appeared significantly less important by the expert cohort. There were no further significant differences in the relative preference weights of OxCAP-MH items between the cohorts or according to gender.

**Conclusions:**

Our study indicates significant between-item but limited mental ill-health related heterogeneity in the relative preference weights of the different capability items within the OxCAP-MH. The findings support the future development of preference-based value sets elicited from the general population for comparative economic evaluation purposes.

**Supplementary Information:**

The online version contains supplementary material available at 10.1186/s12955-022-02009-6.

## Introduction

The assessment of outcomes in health intervention and care related studies requires information not only about the identification and measurement of impacts, but also the consideration of how valuable these impacts are to patients or the general public [1]. Outcomes in the area of mental health often go beyond health and incorporate wider impacts [2]. The capability framework, introduced by Amartya Sen in the early 1980s as an alternative to standard utilitarian welfare economics, provides a richer evaluative space beyond health [3]. The core focus of the capability approach is on what individuals are able to be and do in their lives, in other words, what they are capable of [4]. One of the outcome assessment instruments specifically designed to capture different dimensions of well-being within the capability framework in the area of mental health is the self-reported Oxford Capability questionnaire-Mental Health (OxCAP-MH) instrument [2]. The development of the OxCAP-MH was based on the list of ten central capabilities endorsed by Martha Nussbaum in 2003 [5]. This list included life; bodily health; bodily integrity; senses, imagination and thought; emotions; practical reason; affiliation; other species; play; and control over one’s environment. The scoring of the OxCAP-MH currently relies on equal weights across items and the instrument has been used also as a non-preference based outcome measure in economic evaluations so far [6]. There are, however, suggestions in the literature that some capability items may be more important than others in determining someone’s well-being [5, 7]. The weighting of preferences may vary not only between different cultural settings (i.e. regions/countries) and main sociodemographic characteristics (i.e. age, gender), but also across different cohorts influenced by specific insight into or adaptation to an illness [8–10]. It has long been acknowledged in the preference elicitation and value weighting literature that the time spent in a health state may have an influence on the way that state is perceived too [11]. Beside the methods used for valuation, controversies exist concerning who should provide valuations. In principle, preferences or values could be provided by members of the general public, who most often pay for interventions via taxation or social insurance; or by patients, who have lived experience with the health state in question; or by experts, who have relevant scientific or clinical insights [12].

The idea of developing value sets for capability instruments has first appeared in 2008, during the development of the OCAP-18 [13]. However, there is still no consensus on the best method to elicit values and whether capability instruments should be anchored similarly to preference-based health-related quality of life (HRQoL) measures. A recent literature review identified that all studies aiming to develop value sets for capability instruments used the Best–Worst Scaling (BWS) method [14]. These include the UK value sets for the Adult Social Care Outcomes Toolkit (ASCOT), Investigating Choice Experiments Capability Measure for Adults (ICECAP-A), for older people (ICECAP-O) and Supportive Care Measure (ICECAP-SCM) instruments [7, 15–17]. Moreover, a recently published study aimed to establish Austrian preference weights for the ASCOT instrument also used the BWS method [18]. The main reason for this is that BWS elicits values rather than preferences because individuals are not asked to trade one thing for another, but rather only state which option they find most and least important within hypothetical scenarios [15]. Thus, the BWS approach may come closest from all the potentially available methods, which have been used to elicit value sets, that would satisfy Sen's interpretation of the capability approach [3]. Moreover, alternative methods, such as discrete choice experiments (DCEs), could not handle the number of attributes and levels in the OxCAP-MH and may pose problems disentangling independent effects because of inter-item correlations [19].

This study aimed to explore the relative preference weights of the 16 items of the German language version of the OxCAP-MH (Oxford Capability questionnaire-Mental Health) capability instrument and their differences across cohorts with alternative levels of mental ill-health experience.

## Methods

### OxCAP-MH

The study was conducted prospectively in Austria using the German language version of the OxCAP-MH capability wellbeing questionnaire. The OxCAP-MH is a 16-item instrument originally developed for capability wellbeing measurement in the mental health context. Items are rated on a 1–5 Likert scale. The total level sum score assumes equal weights between the different items and is converted using the formula: 100 × [OxCAP-MH total score – minimum score)/range to arrive to a standardised score between 0 and 100. In other words, the total raw score is calculated by multiplying the number of items with the number of levels: 16 × 5 = 80, the minimum score therefore equals 16, whilst the range is 64 (16–80). Higher scores indicate better capabilities [20]. The OxCAP-MH has been so far validated in English [20], German [21, 22], Luganda [23], Hungarian [24] and Chinese [25] languages and used in multiple studies [26–28]. A sample copy of the OxCAP-MH is available at https://healtheconomics.meduniwien.ac.at/downloads/oxcap-mh/, and the full list of items is included in the Additional file 1.

### Best–worst scaling (BWS) experiment

Relative preference weights of the OxCAP-MH capability items were elicited by a Best–Worst Scaling (BWS) experiment where an individual's decision regarding the most and least preferred options in a set of attributes is elicited across repeated hypothetical scenarios that are systematically varied via an experimental design.

The survey questionnaire consisted of the BWS instrument and several sociodemographic questions, including age, gender, education, living environment, marital status and indirect experience with mental disorders. The attributes of the hypothetical scenarios for the BWS were developed from the 16 OxCAP-MH items, in a set of qualitative interviews following pilot testing with seven persons [29, 30]. Item wording adaptation was necessary so that the attribute wordings reflected the correct individual choices and was carried out similar to the attribute development process for DCE studies [29].

The valuation of item weights was based on the object case of the BWS method. The object case is designed to determine the relative importance of the listed attributes (adapted OxCAP-MH items), which have no level, and choice scenarios differ merely in the particular subset of attributes shown [31]. Each participant was presented with 16 hypothetical scenarios with six attributes. This was considered as the optimal, sufficient design based on recommendations from the literature [32] and established in the pilot phase. Respondents were asked to select the most and least important attributes for them as an individual. An example of the choice task is shown in Table 1, and an exemplary questionnaire can be seen in the Additional file 1.

**Table 1:** Exemplary BWS task

Please imagine that you have to choose between the following 6 options based on what are the most important and least important things in your life. Please choose the one most important and the one least important options:		
Most important		Least important
	My health does not limit my daily activities in any way compared to most people of my age	
	I am not assaulted (including sexual and domestic assault)	
	I am able to influence decision affecting my local area	
	I am able to appreciate and value plants, animals and the world of nature	
	I have access to interesting forms of activity (or employment)	
	I am free to use my imagination and to express myself creatively (e.g. through art, literature, music, etc.)	

The 16 items of the German OxCAP-MH, each of them with 5 levels (5^16), would result in 152,587.890,625 potential combinations of items and levels, i.e. capability states. Currently available BWS methods for the weighting of instruments are not able to handle this many hypothetical states because respondents would be presented with a number of hypothetical scenarios that they are not able to cope with. One potential option to solve this problem would be to divide the potential options into blocks, and only present respondents with a manageable number of scenarios. However, this would have required a study participant sample size that was not feasible in practice. We, therefore, concentrated on the preference weights of the 16 items without including levels. Hypothetical scenarios were reduced through Balanced Incomplete Block Design (BIBD) in Sawtooth Software [30, 33]. BIBD means that each item appears the same number of times, and it also forces paired combinations of attributes to appear together [34]. Part of the analysis focused on three cohorts of the overall sample which reduced the sample size, hence, response efficiency was increased by developing three blocks of the BWS questionnaire with the Paper-and-Pencil module of Sawtooth Software’s Lighthouse Studio 9.13.1, based on 1000 iterations. All three versions were equally distributed within each cohort of respondents and randomly assigned to them.

### Participants and data collection

The study included three cohorts of participants from Austria: (1) psychiatric patients (direct mental ill-health experience), (2) (mental) healthcare experts (indirect mental ill-health experience), and 3) primary care patients with no mental ill-health experience. All participants had to be between 18 and 80 years of age and able and willing to complete the questionnaire. Sufficient intellectual capacities and German language skills were judged by the recruiting team. The minimum sample size estimate of 50 in each cohort, i.e. 150 respondents in total, allowed differences between means of a magnitude at least 0.57 within-group standard deviations to be detected with 80% power at a two-sided significance level of 5% and was similar to previous relevant studies that had aimed to develop weights or value sets to capability wellbeing questionnaires [7, 15, 17, 35].

Similar to some other elicitation studies (e.g. [16, 36]), convenience sampling was used. Mental health patients were approached in the relevant health care facilities by their treating health care professionals in the Department of Psychiatry and Psychotherapy, Clinical Division of Social Psychiatry at the Medical University of Vienna. Persons attending primary care appointments were approached in the waiting areas of the collaborating Primary Care Centres in Vienna. (Mental) health experts were recruited through collaborating professional contacts at the Medical University of Vienna. Participants were recruited between January and May 2020. The study received approval from the Medical University of Vienna (EK Medizinische Universität Wien: 1779/2019). People were first given a participant information sheet and received the BWS questionnaire after they provided consent to participation in the study. The survey was paper-based and self-completed. Data were pseudo-anonymised for further analysis.

### Statistical analysis

The statistical analysis aimed to estimate weights to the OxCAP-MH items across different cohorts. Sociodemographic characteristics of the respondents were summarised as mean values by cohorts. The weights for the items were calculated by Hierarchical Bayes (HB) estimation using Sawtooth Software’s analysis tool. HB estimation was employed because its calculation is based on individual respondents and consequently enables segmentation by cohorts. Instead of estimating each respondent’s utilities individually, the algorithm estimates how different the respondent’s utilities are from the other respondents in the study [37]. Hence, the HB estimation is based on the probability that a respondent selects a specific concept in a choice task given a specific set of preferences and the probability that the respondent’s preferences are consistent with the patterns of the preferences observed in all other respondents [37]. HB estimation can yield reliable individual best–worst values even when the number of responses per participant is small [38]. The mean relative importance score (RIS) was calculated for each item based on HB estimation [39]. An individual fit statistic (root likelihood) per respondent below 0.2 was used to identify inconsistent responders following guidance from Orme [40]. A lower value would be an indication of purely random responses to the choice task. Differences between the RIS scores calculated by HB estimation were explored in a graphical presentation and tables including rank orders of the items. The rank order analysis based on HB estimation was repeated for cohorts, testing the differences across groups by Kruskal–Wallis equality of populations rank tests. Furthermore, to quantify linear association between variables, Pearson correlation coefficients between the RIS scores of the items for the full cohort were calculated and visualised by means of a heatmap.

Finally, multivariable linear regression analyses were conducted to explore the relative adjusted importance of the items across cohorts. The continuous RIS of each item was regressed on binary group indicators, whilst adjusting for gender and cohort. Other demographic variables (e.g., age, profession) were not considered as adjustments as they were main attributes of the cohorts' characteristics. Robust standard errors to account for violations of model assumptions and the implicit correlation of the outcome variables were obtained using the Jackknife method. It means that each participant was omitted in turn, the HB scores were re-estimated and the regressions of the HB scores on the covariates were re-fitted. From the regression coefficients of these 16 (items) $\times$ 158 (participants) analyses, robust variances of regression coefficients were obtained by the following formula:

$${\widehat{v}}_{kj}=\frac{n-1}{n}\sum_{i=1}^{n}({\stackrel{\sim }{\beta }}_{kj}^{\left(-i\right)}-{\widehat{\beta }}_{kj}{)}^{2},$$

where ${\widehat{\beta }}_{kj}$ denotes the regression coefficient of covariate $j$ for item $k$ (where j and k are indices for covariate and item) from the full sample; and ${\stackrel{\sim }{\beta }}_{kj}^{(-i)}$ denotes the corresponding estimated regression coefficients obtained from re-estimating the HB scores after omitting participant $i$.

The standard errors were calculated as ${\widehat{s}}_{kj}=\sqrt{{\widehat{v}}_{kj}}$, and p-values for testing the hypothesis ${\beta }_{kj}=0$ were obtained by comparing ${\widehat{\beta }}_{kj}/{\widehat{s}}_{kj}$ to a t-distribution with $n-J-1$ degrees of freedom, $J$ being the number of regression coefficients, i.e. four.

The HB estimation and count analysis were performed in Sawtooth Software, the regression analyses were calculated using R version 4.0.2 [41] and all other analyses were conducted with STATA Version 16 [42]. A two-sided significance level of $\alpha =0.05$ was considered to indicate statistical significance in all analyses, unless stated otherwise. P-values were not corrected for multiple testing, but all performed comparisons were reported.

### Indicative preference weight set development

An indicative preference weight set for the German OxCAP-MH was developed for Austria by a linear transformation of values based on HB estimation across all three cohorts. Since each of the 16 OxCAP-MH items has five levels and the assumption was that they carry proportionately constant weights, i.e. their contribution to the overall preference weight was assumed to be equally distributed. Worst capability levels carried the multiplication factor of 0, best capability levels the multiplication factor of 1, and those in-between the multiplication factors of 0.25, 0.5, 0.75, respectively. Similar to other capability instruments, the proposed indicative preference weight set of the OxCAP-MH instrument is anchored on a scale of 0 (no capability) to 1 (full capability).

## Results

### Participant characteristics

**Fig. 1:**
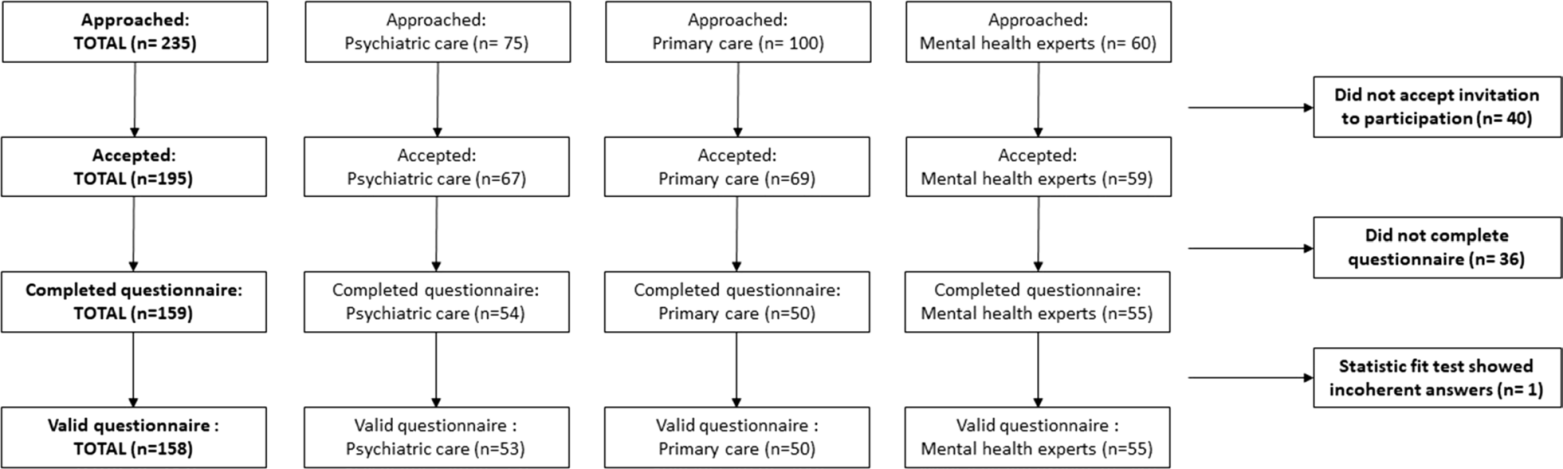
Overview of recruitment strategy and inclusion of participants

Initially, 235 people were approached, with response rates of ca. 90% for psychiatric patients, nearly 100% for (mental) healthcare experts, and ca. 70% for primary care attendees. In total, 195 persons agreed to participate, out of whom 159 participants fully completed the BWS questionnaire and was initially included in the analysis as shown in Fig. 1.

In a subsequent step, one psychiatric patient had to be excluded because the calculated HB fit statistic was under 0.2, and it was therefore assumed that the responses of this participant were provided by chance. Table 2 provides further details on participant characteristics of the final included 158 participants.

**Table 2:** Characteristics of participants

Mean (SD) or %	All respondents	Psychiatric patients	General population	Mental health experts
	N = 158	n = 53	n = 50	n = 55
Age				
18–29	83 (52%)	21 (40%)	22 (44%)	40 (73%)
30–49	51 (32%)	20 (38%)	21 (42%)	10 (18%)
50–64	20 (13%)	9 (17%)	6 (12%)	5 (9%)
65 and above	4 (3%)	3 (6%)	1 (2%)	0 (0%)
Gender				
Female	94 (59%)	32 (60%)	32 (64%)	30 (55%)
Male	64 (41%)	21 (40%)	18 (36%)	25 (45%)
Education				
Primary	15 (9%)	8 (15%)	7 (14%)	–
Secondary	40 (25%)	24 (45%)	16 (32%)	–
Higher education	103 (65%)	21 (40%)	27 (54%)	55 (100%)
Living environment				
City	133 (84%)	43 (81%)	41 (82%)	49 (89%)
Small town	9 (6%)	4 (8%)	5 (10%)	–
Village	15 (9%)	5 (9%)	4 (8%)	6 (11%)
Remote area	1 (1%)	1 (2%)	–	–
Marital status				
Single	92 (58%)	30 (57%)	21 (42%)	41 (76%)
Married or cohabitating	53 (34%)	18 (34%)	25 (50%)	10 (18%)
Divorced or separated	13 (8%)	5 (9%)	4 (8%)	4 (7%)
Number of children (n = 103)				[no information]
No children	65 (63%)	37 (70%)	28 (43%)	
1 child	20 (19%)	9 (17%)	11 (55%)	
2 children	14 (14%)	5 (9%)	9 (60%)	
3 or more children	4 (4%)	2 (4%)	2 (50%)	
Mental health problems (n = 103)				
Yes	58 (56%)	53 (100%)	5 (10%)	
No	45 (44%)	-	45 (90%)	
Physical problems (n = 103)				
Yes	23 (22%)	19 (36%)	4 (8%)	
No	80 (78%)	34 (64%)	46 (92%)	
Indirect experience with mental disorders (professional or personal)				
Yes	96 (61%)	23 (43%)	18 (36%)	55 (100%)
No	62 (39%)	30 (57%)	32 (64%)	–

### Relative importance of items

**Fig. 2:**
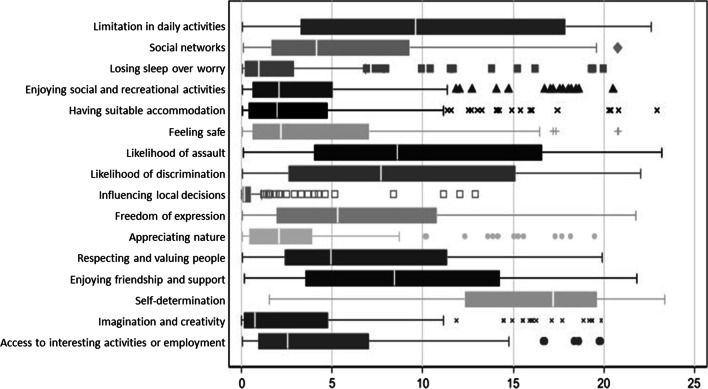
Relative Importance Scores of OxCAP-MH items calculated by Hierarchical Bayes estimation (n = 158). White vertical line = median; box = interquartile range; ‘whiskers’ extending to max 1.5 interquartile ranges outside the box; any values outside the whisker are depicted individually

Figure 2 illustrates the RIS of the items calculated by HB estimation and by means of box plots. The mean RIS scores for the full cohort ranged from 0.76 to 15.72. The RIS based on HB estimation indicate very strong relative preference weights for the items *Self-determination* and *Limitation in daily activities*. The RIS also suggest that *Influencing local decisions* and *Losing sleep over worry* are regarded as the least important items.

More detailed information about the Mean Relative Importance Scores and Standard Deviations of OxCAP-MH items calculated by Hierarchical Bayes estimation can be found in the Additional file 1.

Table 3 provides a rank order of mean RIS scores attributed to the individual OxCAP-MH items calculated by both HB estimation and count analysis, with links to Nussbaum’s ten central human capabilities.

**Table 3:** Rank orders per item using different analysis methods

Item number	Question number	Item	Central human capabilities	HB estimation
1	1	Limitation in daily activities	Bodily health	8
2	2	Social networks	Affiliation	5
3	3	Losing sleep over worry	Emotions	13
4	4	Enjoying social and recreational activities	Play	6
5	5	Having suitable accommodation	Bodily health	14
6	6	Feeling safe	Bodily integrity	7
7	7	Likelihood of assault	Bodily integrity	15
8	8	Likelihood of discrimination	Affiliation	11
9	9a	Influencing local decisions	Control over one's environment	9
10	9b	Freedom of expression	Senses, imagination & thought	3
11	9c	Appreciating nature	Species	1
12	9d	Respecting and valuing people	Affiliation	10
13	9e	Enjoying friendship and support	Emotions	12
14	9f	Self-determination	Practical reason	2
15	9 g	Imagination and creativity	Senses, imagination & thought	4
16	9 h	Access to interesting activities or employment	Control over one's environment	16

**Fig. 3:**
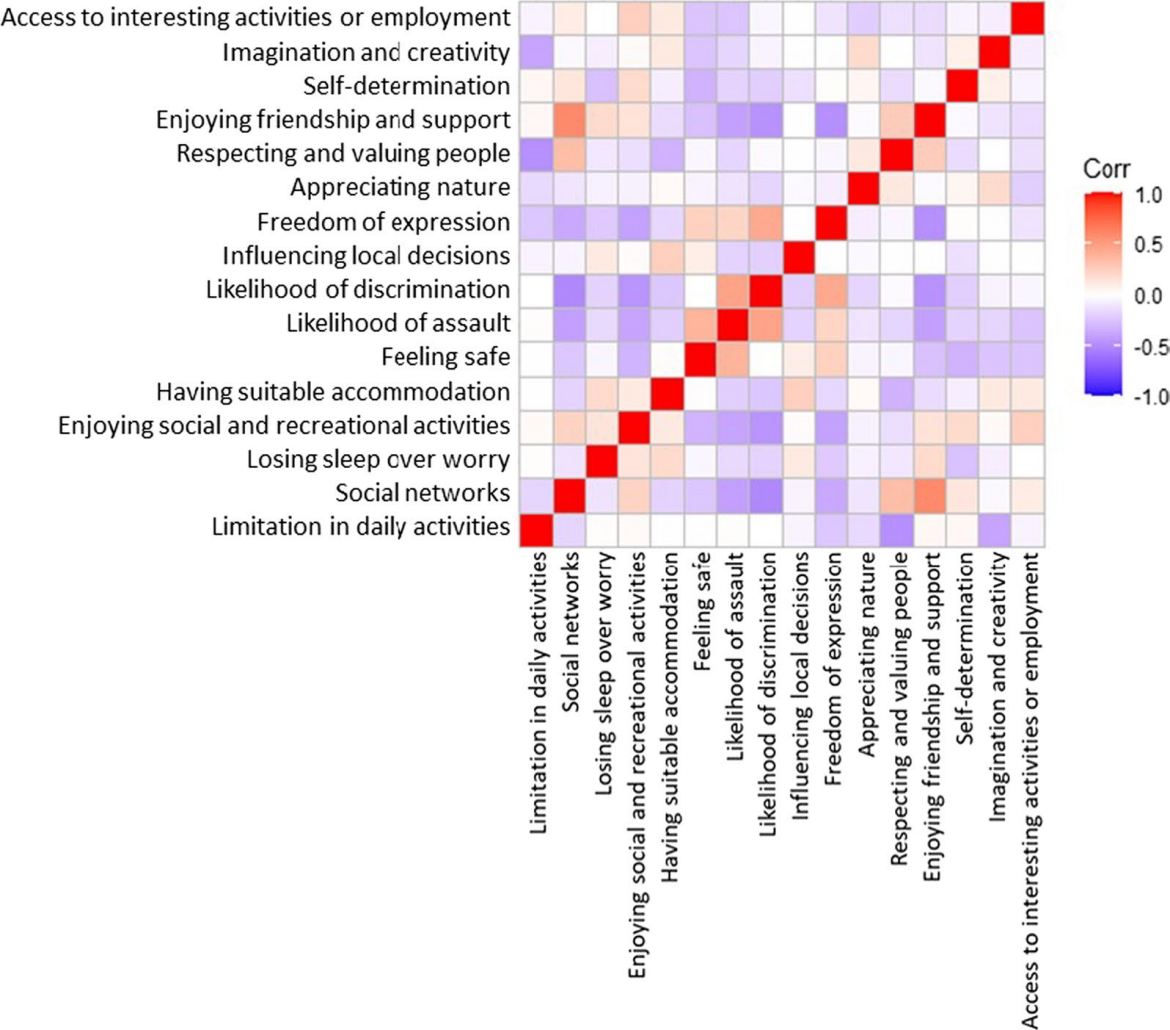
Heatmap of pairwise Pearson correlation coefficients between OxCAP-MH items

Figure 3 provides a heatmap of the correlation between the items, indicating that some items are strongly correlated including: *Social networks* with *Enjoying friendship and support*; *Likelihood of assault* with *Likelihood of discrimination*; and *Likelihood of discrimination* with *Freedom of expression*.

### Analysis by cohorts with different mental ill-health experience and gender

The rank orders and observed mean RIS scores for each of the three cohorts revealed that the mean RIS per cohort significantly differs in eight out of the 16 OxCAP-MH items (Table 4). This is also visible on the graphical presentation of mean RIS estimates by cohort (see Additional file 1).

**Table 4:** Rank order (1 = most important, 16 = least important) and observed mean Relative Importance Scores for each cohort (n = 158)

Item	Question number	Label	Cohort (rank; RIS)			
			Primary care patients	Psychiatric patients	Mental health experts	p value*
			*n* = *50*	*n* = *53*	*n* = *55*	
1	1	Limitation in daily activities	2 (11.99)	2 (9.44)	3 (10.11)	0.281
2	2	Social networks	9 (4.59)	7 (6.72)	8 (6.26)	0.180
3	3	Losing sleep over worry	14 (3.08)	15 (2.36)	14 (2.06)	0.210
4	4	Enjoying social and recreational activities	13 (3.09)	10 (4.81)	11 (3.28)	**0.028**
5	5	Having suitable accommodation	11 (3.68)	8 (5.96)	15 (1.98)	**0.000**
6	6	Feeling safe	8 (5.07)	14 (3.04)	10 (5.11)	**0.002**
7	7	Likelihood of assault	3 (10.00)	4 (8.04)	2 (11.67)	**0.019**
8	8	Likelihood of discrimination	4 (9.02)	5 (7.21)	4 (9.87)	0.091
9	9a	Influencing local decisions	16 (0.64)	16 (0.75)	16 (0.88)	**0.013**
10	9b	Freedom of expression	6 (7.90)	12 (4.64)	6 (7.77)	**0.005**
11	9c	Appreciating nature	12 (3.50)	13 (3.80)	12 (2.38)	0.194
12	9d	Respecting and valuing people	7 (6.54)	6 (7.15)	7 (6.95)	0.851
13	9e	Enjoying friendship and support	5 (8.38)	3 (9.40)	5 (9.24)	0.718
14	9f	Self-determination	1 (15.80)	1 (16.25)	1 (15.15)	0.271
15	9 g	Imagination and creativity	10 (3.71)	11 (4.69)	13 (2.08)	**0.039**
16	9 h	Access to interesting activities or employment	15 (3.01)	9 (5.74)	9 (5.22)	**0.007**

Relative Importance Scores (based on HB estimation) in brackets; p values ≤ 0.05 are in bold; *Kruskal–Wallis equality-of-populations rank test

The regression coefficients with robust standard errors and p-values presented in Table 5 described the gender-adjusted difference in expected RIS between the cohorts and the cohort-adjusted difference between males and females. This analysis revealed that *Freedom of expression* was rated significantly less important by psychiatric patients than by the other two cohorts, given that the adjusted difference in RIS was 3.25 units, which is approximately half of the overall mean RIS for this item. Moreover, *Having suitable accommodation* appeared significantly less important for the expert cohort than for primary care patients with no mental ill-health experience. The difference for this item between psychiatric patients and experts was even larger in terms of the regression coefficient, but with a larger standard error. There were no further significant differences in the relative preference weights of OxCAP-MH items between the cohorts or according to gender.

**Table 5:** Multivariate regression analysis per item with robust standard errors (n = 158)

Item number	Question number	Label	Mean (SD)	Male vs female	Psychiatric patients vs general population	Mental health experts vs. general population	Constant
1	1	Limitation in daily activities	10.48 (7.43)	0.97 (4.71)	− 2.58 (3.97)	− 1.97 (1.75)	11.64 (1.35)
2	2	Social networks	5.89(5.14)	0.21 (1.99)	2.12 (1.82)	1.65 (1.05)	4.52 (0.82)
3	3	Losing sleep over worry	2.48 (3.84)	0.68 (3.45)	− 0.74 (1.22)	− 1.08 (1.56)	2.83 (2.30)
4	4	Enjoying social and recreational activities	3.73 (4.78)	1.50 (4.58)	1.67 (1.47)	0.04 (0.98)	2.55 (2.39)
5	5	Having suitable accommodation	3.85(5.04)	1.39 (3.11)	2.23 (2.11)	− 1.83 (0.80)*	3.18 (1.96)
6	6	Feeling safe	4.41(4.97)	− 1.35 (4.48)	− 1.98 (1.35)	0.16 (1.78)	5.56 (1.91)
7	7	Likelihood of assault	9.92(6.71)	− 2.52 (4.77)	− 1.87 (3.34)	1.91 (1.44)	10.90 (3.25)
8	8	Likelihood of discrimination	8.71(6.65)	− 0.11 (1.44)	− 1.80 (3.52)	0.86 (1.63)	9.06 (1.93)
9	9a	Influencing local decisions	0.76 (1.92)	0.10 (0.98)	0.11 (0.33)	0.23 (0.46)	0.60 (0.42)
10	9b	Freedom of expression	6.76(5.75)	− 0.28 (1.31)	− 3.25 (1.60)*	− 0.11 (1.36)	8.00 (1.27)
11	9c	Appreciating nature	3.22(4.13)	− 0.87 (2.24)	0.33 (1.19)	− 1.04 (1.57)	3.82 (1.35)
12	9d	Respecting and valuing people	6.89(5.58)	− 0.37 (2.58)	0.62 (2.12)	0.44 (1.95)	6.67 (1.57)
13	9e	Enjoying friendship and support	9.02 (6.10)	− 1.04 (1.99)	1.05 (3.03)	0.95 (1.37)	8.76 (1.19)
14	9f	Self-determination	15.72 (4.74)	0.01 (0.99)	0.45 (1.17)	− 0.65 (1.27)	15.80 (0.97)
15	9 g	Imagination and creativity	3.47 (5.34)	1.37 (2.08)	0.93 (2.84)	− 1.75 (1.09)	3.21 (1.83)
16	9 h	Access to interesting activities or employment	4.70(5.03)	0.32 (1.07)	2.72 (1.39)	2.19 (2.00)	2.90 (0.99)

Standard errors in parentheses; ^*^p < 0.05, ^**^p < 0.01, ^***^p < 0.001

### Preference weight set

Table 6 provides an indicative preference weight set that could be used as current best proxy to calculate individual scores for different capability states in economic evaluations [43].

**Table 6:** Preference weights of the OxCAP-MH items and their levels (n = 158)

Item number	Question number	Label	Answer level 1	Answer level 2	Answer level 3	Answer level 4	Answer level 5
1	1	Limitation in daily activities	0.0000	0.0262	0.0524	0.0786	0.1048
2*	2	Social networks	0.0589	0.0442	0.0294	0.0147	0.0000
3	3	Losing sleep over worry	0.0000	0.0062	0.0124	0.0186	0.0248
4*	4	Enjoying social and recreational activities	0.0373	0.0280	0.0187	0.0093	0.0000
5	5	Having suitable accommodation	0.0000	0.0096	0.0192	0.0289	0.0385
6*	6	Feeling safe	0.0441	0.0330	0.0220	0.0110	0.0000
7	7	Likelihood of assault	0.0000	0.0248	0.0496	0.0744	0.0992
8	8	Likelihood of discrimination	0.0000	0.0218	0.0435	0.0653	0.0871
9*	9a	Influencing local decisions	0.0076	0.0057	0.0038	0.0019	0.0000
10*	9b	Freedom of expression	0.0676	0.0507	0.0338	0.0169	0.0000
11*	9c	Appreciating nature	0.0322	0.0241	0.0161	0.0080	0.0000
12*	9d	Respecting and valuing people	0.0689	0.0517	0.0344	0.0172	0.0000
13*	9e	Enjoying friendship and support	0.0902	0.0677	0.0451	0.0226	0.0000
14*	9f	Self-determination	0.1572	0.1179	0.0786	0.0393	0.0000
15*	9 g	Imagination and creativity	0.0347	0.0260	0.0174	0.0087	0.0000
16*	9 h	Access to interesting activities or employment	0.0470	0.0352	0.0235	0.0117	0.0000

^*^Items 2, 4, 6, 9, 10, 11, 12, 13, 14, 15 and 16 are reversed coded. For these items, Answer level 1 represents highest level of capabilities and Answer level 5 represents highest level of capabilities

## Discussion

This study is the first to explore the relative preference weights of the items of the OxCAP-MH, a capability well-being instrument that was originally developed for outcome measurement in this context, and the impact of mental ill-health experience on these. As opposed to other capability instruments with existing preference weight or value sets such as the ASCOT [17], ICECAP-A [7], ICECAP-O [15] and ICECAP-SCM [35], the high number of items posed a major challenge in case of the OxCAP-MH. Hence, this study only focused on the items without incorporating preferences related to levels. The overall study sample was balanced for gender and for differing mental ill-health experiences between the cohorts.

The results confirmed significantly different preference weights attributed to the 16 items of the OxCAP-MH. The RIS scores for the full cohort ranged from 0.76 to 15.72 and indicated very strong relative preference weights for *Self-determination* and *Limitation in daily activities*, suggesting that these concepts have large spans within the ‘capability space’ covered by the OxCAP-MH. The relatively high importance of these items also demonstrates that respondents placed a much higher emphasis on individual aspects of capabilities than on community-based aspects, including *Influencing local decisions*, in the Austrian context [18]. The most important items of the OxCAP-MH are those related to Nussbaum’s practical reasoning and overall health capabilities, whilst aspects related to emotions and control over one’s environment are regarded significantly less important. These results are in line with the findings of the BWS experiment eliciting preference weights for the ASCOT instrument for service users in Austria. Hajji et al. found that the most important choices were related to being meaningfully occupied during the day and having control over daily life, whilst the least important choices were associated with dignity and social participation [18]. These findings are also in line with the results of the UK preference weighting study of the ASCOT [17]. Conversely, the development of the initial UK value set of ICECAP-A in 2013 found that attachment and stability had slightly higher contribution to the capability space then the items of autonomy, achievement and enjoyment. This suggests that the different capability instruments either elicit different aspects of capabilities, or the actual wording of questions and the cultural context play a more significant role than previously thought.

With regard to differences across the cohorts, we could not find evidence that mental ill-health experience or gender were significantly associated with the relative importance of the OxCAP-MH items. While rank analysis revealed some differences across the cohorts, this was not to a significant extent. The magnitudes of the regression coefficients with respect to the mean values of the OxCAP-MH item scores were relatively small across cohorts, apart from the *Having suitable accommodation* and *Freedom of expression* items. These findings suggest that potential future value sets for the OxCAP-MH elicited from the general population are likely to represent well also the direct preferences of people with lived experience of mental ill-health. These results somewhat contradict the findings that patient preferences differ from preferences derived from the general population for the EQ-5D-5L items [10]. In the study by Ludwig et al., 2021, patients in the EQ-5D-5L study gave more importance to mobility, self-care, or usual activities and less importance to pain/discomfort and anxiety/depression compared to the general population [10]. The differential findings between the two studies could be explained by the specific perception of mental health itself and the difference between health-related quality of life and capabilities as evaluative spaces.

Our study is unique by explicitly exploring the impact of ill-health experience on preferences in the context of a capability wellbeing instrument. Moreover, the study is based on a thoroughly designed BWS experiment and sophisticated statistical analysis. This takes into account the multivariate distribution with implicit correlation of outcomes, and robustness against model misspecification and violation of assumptions of linear regression, which probably implied larger estimated standard errors.

The interpretation of the study results, however, require the consideration of its limitations. One of the main limitations is the relatively small sample size. A larger number of observations would have strengthened the robustness of the statistical analysis, and also it would have enabled the formation of bigger groups for subgroup analyses, including latent class estimation. Moreover, we adjusted for gender, but not for other demographic variables because they were intrinsic to the definition of the population, for instance, in terms of experts’ age. This meant that the analysis compared the items between population cohorts in a simple form and did not artificially equalise their characteristics, which would have caused over-adjustment and could have induced bias. A further limitation is that the sample is not representative of the Austrian population because it is generally younger and all respondents live in or around Vienna. In the future, the current methods could be expanded and be developed into a full preference weight set using a representative Austrian general population sample. Finally, the research focused only on the capability items of the OxCAP-MH themselves and not their levels due to the focus of our research and the high number of possible item-level combinations which would have proven challenging to incorporate. The assumption that levels carry proportionately constant weights may prove problematic for an actual value set development. Similar BWS studies developing weights for capability instruments, including the ICECAP-A and the Carer Experience Scale, found that greater value was placed on differences between the bottom and middle levels of items than between the middle and top levels [7, 44]. Future preference weight set developments should address the issue of preferences related to levels. The indicative preference weight set could be used as current best proxy to calculate individual scores for different capability states in economic evaluations [42].

## Conclusion

This study found no evidence that preference weights for the items of the mental health-specific OxCAP-MH capability well-being instrument could not be elicited from alternative population cohorts with differing mental ill-health experience. Interim, the current preference weight set can be seen as the best proxy for calculating individual scores for different capability states as defined by the OxCAP-MH in economic evaluations. Our approach can also serve as an example for other BWS preference elicitation studies for more complex patient-reported outcome measures (PROMs) in order to be used for cost-effectiveness analyses.

## Supplementary Information

**Additional file 1. Appendix 1:** List of OxCAP-MH items, questions and attributes included in the BWS task. **Appendix 2:** Mean Relative Importance Scores and Standard Deviations of OxCAP-MH domains calculated by hierarchical Bayes estimation. **Appendix 3:** Sample BWS questionnaire (translated from German). **Appendix 4:** Mean Relative Importance Scores (Hierarchical Bayes estimates) by cohort (n=158)

## Data Availability

The datasets used and analysed during the current study are available from the corresponding author on reasonable request.
