# Supplementary material for: Does the relative importance of the OxCAP-MH’s capability items differ according to mental ill-health experience?
Source: Health Qual Life Outcomes. 2022 Jun 24;20:99. doi: 10.1186/s12955-022-02009-6 (PMC9233329; doi:10.1186/s12955-022-02009-6)

## Appendix 1: List of OxCAP-MH items, questions and attributes included in the BWS task

| Item | OxCAP-MH abbreviation | OxCAP-MH question | Attribute in the BWS task |
|---|---|---|---|
| 1 | Limitation in daily activities | Does your health in any way limit your daily activities, compared to most people of your age? | My health does not limit my daily activities in any way compared to most people of my age. |
| 2 | Social networks | Are you able to meet socially with friends or relatives? | I am able to meet socially with friends or relatives. |
| 3 | Losing sleep over worry | In the past 4 weeks, how often have you lost sleep over worry? | I do not lose sleep over worry. |
| 4 | Enjoying social and recreational activities | In the past 4 weeks, how often have you been able to enjoy your recreational activities? | I am able to enjoy my recreational activities. |
| 5 | Having suitable accommodation | How suitable or unsuitable is your accommodation for your current needs? | My accommodation is suitable for my needs. |
| 6 | Feeling safe | Please indicate how safe you feel walking alone in the area near your home: | I feel safe walking alone in the area near my home. |
| 7 | Likelihood of assault | Please indicate how likely you believe it to be that you will be assaulted in the future (including sexual and domestic assault): | I am not assaulted (including sexual and domestic assault). |
| 8 | Likelihood of discrimination | How likely do you think it is that you will experience discrimination? | I do not experience discrimination. |
| *[8a]* | | *[On what grounds do you think it is likely that you will be discriminated against?]* | |
| 9a | Influencing local decisions | I am free to influence decisions affecting my local area. | I am able to influence decision affecting my local area. |
| 9b | Freedom of expression | I am free to express my views, including political and religious views. | I am free to express my views, including political and religious views. |
| 9c | Appreciating nature | I am able to appreciate and value plants, animals and the world of nature. | I am able to appreciate and value plants, animals and the world of nature. |
| 9d | Respecting and valuing people | I am able to respect, value and appreciate people around me | I am able to respect, value and appreciate people around me. |
| 9e | Enjoying friendship and support | I find it easy to enjoy the love, care and support of my family and/or friends. | I find it easy to enjoy the love, care and support of my family and/or friends. |
| 9f | Self-determination | I am free to decide for myself how to live my life. | I am free to decide for myself how to live my life. |
| 9g | Imagination and creativity | I am able to use my imagination and to express myself creatively (e.g. through art, literature, music, etc.). | I am free to use my imagination and to express myself creatively (e.g. through art, literature, music, etc.). |
| 9h | Access to interesting activities or employment | I have access to interesting forms of activity (or employment). | I have access to interesting forms of activity (or employment). |

**Appendix 2: Mean Relative Importance Scores and Standard Deviations of OxCAP-MH domains calculated by Hierarchical Bayes estimation**

| Domain | Question Number | Label | Full cohort (n=158) | | GP patients (n=50) | | Psychiatric patients (n=53) | | Mental health experts (n=55) | |
|---|---|---|---|---|---|---|---|---|---|---|
| | | | Mean | (SD) | Mean | (SD) | Mean | (SD) | Mean | (SD) |
| 1 | 1 | Limitation in daily activities | 10.48 | (7.43) | 11.99 | (7.72) | 9.44 | (7.75) | 10.11 | (6.74) |
| 2 | 2 | Social networks | 5.89 | (5.14) | 4.59 | (4.24) | 6.72 | (5.71) | 6.26 | (5.16) |
| 3 | 3 | Losing sleep over worry | 2.48 | (3.84) | 3.08 | (4.26) | 2.36 | (3.64) | 2.06 | (3.62) |
| 4 | 4 | Enjoying social and recreational activities | 3.73 | (4.78) | 3.09 | (4.20) | 4.81 | (5.17) | 3.28 | (4.78) |
| 5 | 5 | Having suitable accommodation | 3.85 | (5.04) | 3.68 | (4.48) | 5.96 | (6.38) | 1.98 | (2.90) |
| 6 | 6 | Feeling safe | 4.41 | (4.97) | 5.07 | (5.17) | 3.04 | (4.47) | 5.11 | (5.05) |
| 7 | 7 | Likelihood of assault | 9.92 | (6.71) | 10.00 | (6.47) | 8.04 | (6.25) | 11.67 | (6.97) |
| 8 | 8 | Likelihood of discrimination | 8.71 | (6.65) | 9.02 | (6.46) | 7.21 | (6.21) | 9.87 | (7.06) |
| 9 | 9a | Influencing local decisions | 0.76 | (1.92) | 0.64 | (1.12) | 0.75 | (1.38) | 0.88 | (2.77) |
| 10 | 9b | Freedom of expression | 6.76 | (5.75) | 7.90 | (6.07) | 4.64 | (4.59) | 7.77 | (5.97) |
| 11 | 9c | Appreciating nature | 3.22 | (4.13) | 3.50 | (4.34) | 3.80 | (4.62) | 2.38 | (3.30) |
| 12 | 9d | Respecting and valuing people | 6.89 | (5.58) | 6.54 | (5.49) | 7.15 | (5.86) | 6.95 | (5.49) |
| 13 | 9e | Enjoying friendship and support | 9.02 | (6.10) | 8.38 | (5.84) | 9.40 | (6.09) | 9.24 | (6.39) |
| 14 | 9f | Self-determination | 15.72 | (4.74) | 15.80 | (4.68) | 16.25 | (4.85) | 15.15 | (4.72) |
| 15 | 9g | Imagination and creativity | 3.47 | (5.34) | 3.71 | (5.57) | 4.69 | (6.09) | 2.08 | (3.94) |
| 16 | 9h | Access to interesting activities or employment | 4.70 | (5.03) | 3.01 | (4.05) | 5.74 | (5.56) | 5.22 | (4.99) |

## Appendix 3: Sample BWS questionnaire (translated from German)

Please imagine that you have to select one of the following six options, based on what is the personally most and least important aspect of life to you. Please select both the most and least important options.

(1 of 16)

| Most important | | Least important |
|---|---|---|
| | My health does not limit my daily activities in any way compared to most people of my age. | |
| | I am not assaulted (including sexual and domestic assault). | |
| | I am able to influence decision affecting my local area. | |
| | I am able to appreciate and value plants, animals and the world of nature. | |
| | I have access to interesting forms of activity (or employment). | |
| | I am free to use my imagination and to express myself creatively (e.g. through art, literature, music, etc.). | |

Please imagine that you have to select one of the following six options, based on what is the personally most and least important aspect of life to you. Please select both the most and least important options.

(2 of 16)

| Most important | | Least important |
|---|---|---|
| | I am not assaulted (including sexual and domestic assault). | |
| | My health does not limit my daily activities in any way compared to most people of my age. | |
| | I find it easy to enjoy the love, care and support of my family and/or friends. | |
| | I do not experience discrimination (based on the following reasons: nationality/ethnicity, gender, religion, sexual orientation, age, health status, or disability – including mental health). | |
| | I am able to respect, value and appreciate people around me. | |
| | I do not lose sleep over worry. | |

Please imagine that you have to select one of the following six options, based on what is the personally most and least important aspect of life to you. Please select both the most and least important options.

(3 of 16)

| Most important | | Least important |
|---|---|---|
| | I am free to use my imagination and to express myself creatively (e.g. through art, literature, music, etc.). | |
| | I have access to interesting forms of activity (or employment). | |
| | I am able to respect, value and appreciate people around me. | |
| | I am free to express my views, including political and religious views. | |
| | I am able to enjoy my recreational activities. | |
| | I feel safe walking alone in the area near my home. | |

Please imagine that you have to select one of the following six options, based on what is the personally most and least important aspect of life to you. Please select both the <u>most</u> and <u>least</u> important options.

(4 of 16)

| Most important | | Least important |
|---|---|---|
| | I do not experience discrimination (based on the following reasons: nationality/ethnicity, gender, religion, sexual orientation, age, health status, or disability – including mental health). | |
| | I do not lose sleep over worry. | |
| | I have access to interesting forms of activity (or employment). | |
| | I feel safe walking alone in the area near my home. | |
| | My accommodation is suitable for my needs. | |
| | I am able to meet socially with friends or relatives. | |

Please imagine that you have to select one of the following six options, based on what is the personally most and least important aspect of life to you. Please select both the <u>most</u> and <u>least</u> important options.

(5 of 16)

| Most important | | Least important |
|---|---|---|
| | I do not lose sleep over worry. | |
| | I am free to use my imagination and to express myself creatively (e.g. through art, literature, music, etc.). | |
| | My health does not limit my daily activities in any way compared to most people of my age. | |
| | My accommodation is suitable for my needs. | |
| | I am free to express my views, including political and religious views. | |
| | I am free to decide for myself how to live my life. | |

Please imagine that you have to select one of the following six options, based on what is the personally most and least important aspect of life to you. Please select both the <u>most</u> and <u>least</u> important options.

(6 of 16)

| Most important | | Least important |
|---|---|---|
| | I am free to express my views, including political and religious views. | |
| | I find it easy to enjoy the love, care and support of my family and/or friends. | |
| | I am not assaulted (including sexual and domestic assault). | |
| | I feel safe walking alone in the area near my home. | |
| | I am able to appreciate and value plants, animals and the world of nature. | |
| | I do not experience discrimination (based on the following reasons: nationality/ethnicity, gender, religion, sexual orientation, age, health status, or disability – including mental health). | |

Please imagine that you have to select one of the following six options, based on what is the personally most and least important aspect of life to you. Please select both the most and least important options.

(7 of 16)

| Most important | | Least important |
|---|---|---|
| | I feel safe walking alone in the area near my home. | |
| | I am free to decide for myself how to live my life. | |
| | My accommodation is suitable for my needs. | |
| | I find it easy to enjoy the love, care and support of my family and/or friends. | |
| | I am able to respect, value and appreciate people around me. | |
| | I have access to interesting forms of activity (or employment). | |

Please imagine that you have to select one of the following six options, based on what is the personally most and least important aspect of life to you. Please select both the most and least important options.

(8 of 16)

| Most important | | Least important |
|---|---|---|
| | My accommodation is suitable for my needs. | |
| | I feel safe walking alone in the area near my home. | |
| | I am able to enjoy my recreational activities. | |
| | I am free to use my imagination and to express myself creatively (e.g. through art, literature, music, etc.). | |
| | I do not experience discrimination (based on the following reasons: nationality/ethnicity, gender, religion, sexual orientation, age, health status, or disability – including mental health). | |
| | I am able to appreciate and value plants, animals and the world of nature. | |

Please imagine that you have to select one of the following six options, based on what is the personally most and least important aspect of life to you. Please select both the most and least important options.

(9 of 16)

| Most important | | Least important |
|---|---|---|
| | I find it easy to enjoy the love, care and support of my family and/or friends. | |
| | I am able to enjoy my recreational activities. | |
| | I am free to decide for myself how to live my life. | |
| | My health does not limit my daily activities in any way compared to most people of my age. | |
| | I am not assaulted (including sexual and domestic assault). | |
| | I am free to use my imagination and to express myself creatively (e.g. through art, literature, music, etc.). | |

Please imagine that you have to select one of the following six options, based on what is the personally most and least important aspect of life to you. Please select both the <u>most</u> and <u>least</u> important options.

(10 of 16)

| Most important | | Least important |
|---|---|---|
| | I am able to respect, value and appreciate people around me. | |
| | I am able to meet socially with friends or relatives. | |
| | I am free to use my imagination and to express myself creatively (e.g. through art, literature, music, etc.). | |
| | I am free to decide for myself how to live my life. | |
| | I do not experience discrimination (based on the following reasons: nationality/ethnicity, gender, religion, sexual orientation, age, health status, or disability – including mental health). | |
| | I am free to express my views, including political and religious views. | |

Please imagine that you have to select one of the following six options, based on what is the personally most and least important aspect of life to you. Please select both the <u>most</u> and <u>least</u> important options.

(11 of 16)

| Most important | | Least important |
|---|---|---|
| | I am free to express my views, including political and religious views. | |
| | I am able to respect, value and appreciate people around me. | |
| | I am able to meet socially with friends or relatives. | |
| | I do not lose sleep over worry. | |
| | My health does not limit my daily activities in any way compared to most people of my age. | |
| | I am able to influence decision affecting my local area. | |

Please imagine that you have to select one of the following six options, based on what is the personally most and least important aspect of life to you. Please select both the <u>most</u> and <u>least</u> important options.

(12 of 16)

| Most important | | Least important |
|---|---|---|
| | I am able to appreciate and value plants, animals and the world of nature. | |
| | I am able to influence decision affecting my local area. | |
| | I do not lose sleep over worry. | |
| | I have access to interesting forms of activity (or employment). | |
| | I find it easy to enjoy the love, care and support of my family and/or friends. | |
| | I am able to enjoy my recreational activities. | |

Please imagine that you have to select one of the following six options, based on what is the personally most and least important aspect of life to you. Please select both the most and least important options.

(13 of 16)

| Most important | | Least important |
|---|---|---|
| | I am able to influence decision affecting my local area. | |
| | I am free to express my views, including political and religious views. | |
| | I am able to appreciate and value plants, animals and the world of nature. | |
| | I am able to respect, value and appreciate people around me. | |
| | I am not assaulted (including sexual and domestic assault). | |
| | My accommodation is suitable for my needs. | |

Please imagine that you have to select one of the following six options, based on what is the personally most and least important aspect of life to you. Please select both the most and least important options.

(14 of 16)

| Most important | | Least important |
|---|---|---|
| | I am free to decide for myself how to live my life. | |
| | I am able to meet socially with friends or relatives. | |
| | I have access to interesting forms of activity (or employment). | |
| | I am not assaulted (including sexual and domestic assault). | |
| | I do not lose sleep over worry. | |
| | I am able to enjoy my recreational activities. | |

Please imagine that you have to select one of the following six options, based on what is the personally most and least important aspect of life to you. Please select both the most and least important options.

(15 of 16)

| Most important | | Least important |
|---|---|---|
| | I am able to meet socially with friends or relatives. | |
| | I am able to appreciate and value plants, animals and the world of nature. | |
| | I feel safe walking alone in the area near my home. | |
| | I am able to influence decision affecting my local area. | |
| | I am free to decide for myself how to live my life. | |
| | My health does not limit my daily activities in any way compared to most people of my age. | |

Please imagine that you have to select one of the following six options, based on what is the personally most and least important aspect of life to you. Please select both the most and least important options.

(16 of 16)

| Most important | | Least important |
|---|---|---|
| | I am able to enjoy my recreational activities. | |
| | I do not experience discrimination (based on the following reasons: nationality/ethnicity, gender, religion, sexual orientation, age, health status, or disability – including mental health). | |
| | My accommodation is suitable for my needs. | |
| | I am able to meet socially with friends or relatives. | |
| | I am able to influence decision affecting my local area. | |
| | I find it easy to enjoy the love, care and support of my family and/or friends. | |

**Appendix 4: Mean Relative Importance Scores (Hierarchical Bayes estimates) by cohort (n=158)**

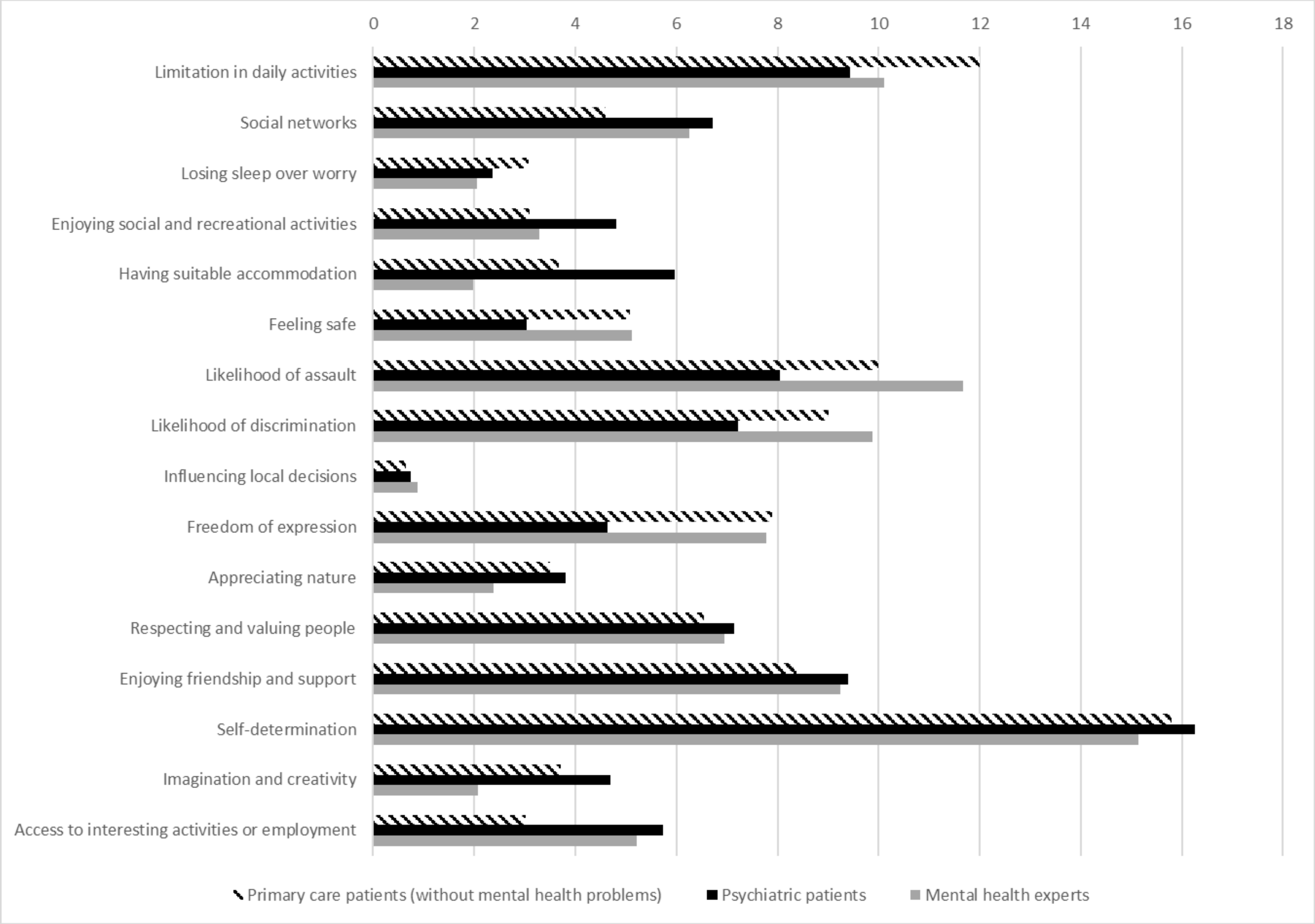

Supplement: Supplementary file 1 — Additional file 1. Appendix 1: List of OxCAP-MH items, questions and attributes included in the BWS task. Appendix 2: Mean Relative Importance Scores and Standard Deviations of OxCAP-MH domains calculated by hierarchical Bayes estimation. Appendix 3: Sample BWS questionnaire (translated from German). Appendix 4: Mean Relative Importance Scores (Hierarchical Bayes estimates) by cohort (n=158) [file 12955_2022_2009_MOESM1_ESM.pdf]
